# Evaluating Postpartum Hemorrhage Transfusion Risk With a Machine Learning Model for Informed Consent: Retrospective Cohort Study

**DOI:** 10.2196/82424

**Published:** 2026-06-11

**Authors:** Jennifer Glance, Jeffrey Arthur Nielson, Albert Bonnema

**Affiliations:** 1 Clinical Informatics Kettering Medical Center Kettering, OH United States; 2 Boonshoft School of Medicine Wright State University Fairborn, OH United States; 3 Heritage College of Osteopathic Medicine Ohio University Athens, OH United States

**Keywords:** machine learning, decision support, ehealth, risk assessment, predictive analytics, clinical decision support, medical informatics, informed consent, postpartum hemorrhage

## Abstract

**Background:**

Postpartum hemorrhage requiring a blood transfusion is a concern for patients and clinicians; its risk and the mode of delivery are important points of discussion before labor. Many high-risk factors associated with postpartum hemorrhage are known prior to delivery and are often unpreventable. Delivery plans are influenced by the patient’s medical history, their preferences, and clinical decision-making. Informed consent regarding known risk factors for postpartum hemorrhage will help guide delivery care plans and mitigate risk. Machine learning models have been used to predict postpartum hemorrhage; however, translation into clinical support tools is challenging. Shared decision-making discussions can be facilitated with machine learning model–based clinical support tools predicting postpartum hemorrhage requiring a transfusion.

**Objective:**

We sought to develop a machine learning model for prediction of postpartum hemorrhage requiring a transfusion. Specifically, we sought to evaluate the model’s accuracy in predicting a patient’s postpartum transfusion risk based on delivery mode, whether labor was induced, and the delivery indications for the purpose of antenatal clinical decision support. Model performance was evaluated on existing structured data and physician-reviewed datasets.

**Methods:**

A 10-year retrospective cohort of 62,521 births in a community health system was sampled. A convenience sample of 1734 patients was analyzed to predict blood transfusion rate based on delivery mode and delivery indications. XGBoost, random forest, and generalized linear models were trained and compared for performance. Datasets were evaluated using the best-performing XGBoost machine learning model. A prototype clinical support app for physician-patient transfusion risk assessment was developed using the best-performing clinically relevant XGBoost model.

**Results:**

A generalized linear model, random forest model, and XGBoost model were evaluated. The XGBoost model was trained with an existing dataset extracted from electronic medical records (n=1734). The area under the curve (AUC) was 0.71, precision-recall receiver operating characteristic curve (PR-ROC) was 0.82, and *F*_1_ score was 0.80. Performance on a physician-reviewed dataset (n=1734) was as follows: AUC=0.705, PR-ROC=0.78, and *F*_1_ score=0.809. Feature importance ranking and prediction were not clinically accurate for the pre-review dataset.

**Conclusions:**

Machine learning models are useful to determine an individual’s postpartum transfusion risk based on clinically variable and potentially modifiable factors, such as delivery mode, whether labor is induced, and delivery indications. In this study, the XGBoost model had a slightly higher AUC on structured data extracted from electronic medical records than the same dataset after physician review (AUC=0.71 and PR-ROC=0.82 vs AUC=0.705 and PR-ROC=0.78), but a slightly lower *F*_1_ score (*F*_1_=0.80 vs *F*_1_=0.809). XGBoost machine learning models trained on clinician-reviewed data can be used to predict postpartum transfusion. Clinically relevant, physician-labeled datasets are important for supervised machine learning model training for use in clinical decision support tools. Further study and external validation are needed prior to clinical use.

## Introduction

Postpartum hemorrhage (PPH) is the leading cause of maternal morbidity in the world and in the United States [1]. The prevalence of blood transfusion following a PPH is 2% to 2.7% [2-5]. The disclosure of transfusion risk with delivery mode and indication is an important aspect of informed consent and shared clinical decision-making [5]. Discussing risk prior to delivery helps physicians and patients understand shared goals and patient preferences for treatment plans. Machine learning has been used to predict transfusion risk for PPH in many studies [4,6-15]; however, the translation to clinical decision support tools has been challenging.

To analyze the association of delivery mode, induction of labor, and delivery indications with PPH requiring a blood transfusion, a machine learning model–based proof-of-concept clinical support application tool was created for Kettering Health, a metropolitan community health system in Dayton, Ohio.

The primary goal of this study was to develop a machine learning model to predict postpartum blood transfusion risk to support a patient-centered approach to care. The secondary goal was to develop a clinical support tool using local data to predict transfusion risk for the purpose of informed consent, shared decision-making, and quality improvement.

## Methods

### Overview

A 10-year retrospective cohort of 62,521 births from January 1, 2015, to May 13, 2025, at Kettering Health was used to obtain a convenience sample of 1734 delivery encounters. The population prevalence of postpartum blood transfusions at Kettering Health was reviewed over the 10-year period and found to be 2% to 2.7%. The sample size was estimated using a 2% population prevalence, a *z* score of 1.96, a 1% margin of error, and 95% CIs. The postpartum transfusion prevalence was also evaluated in 6-month intervals between January 1, 2015, and May 13, 2025, for population distribution. Vaginal delivery occurred in 65% of the population. Delivery mode, delivery indications, and transfusion rates were reviewed. The top 50 cesarean section delivery indications were grouped into 7 categories with medical staff leadership and obstetrician input. Vaginal delivery in structured data is labeled as an “unspecified” indication for a cesarean section. However, indications for cesarean section that are not detailed may also be listed as “unspecified” in the electronic medical record (EMR). Each cesarean indication and vaginal delivery group was reviewed for whether they received or did not receive a transfusion to determine distribution over the 10-year period for quality review. Population race, ethnicity, and age at the time of delivery were not used in model training. Patient age at time of delivery was not calculated for the dataset due to concerns that the delivery date comprises part of an infant’s protected health information. Table 1 reports the characteristics of the total delivery population, and Table 2 reports the characteristics of the study cohort. Labeling of data was performed using existing, structured EMR data (Epic; Epic Systems) and a manual chart review by a physician. Patients with prior deliveries, prior transfusions, and prior cesarean sections were present in the convenience sample. Multiple births for the same patient’s delivery date were normalized. Breech presentation was excluded from the convenience dataset as the primary indication for cesarean delivery.

Structured data were taken from the EMR. Delivery mode was a binary variable with absence of a cesarean section labeled as a vaginal delivery. Delivery indications were grouped into 7 categories: elective, failed induction of labor, macrosomia, medical or surgical indication, other, repeat, and vaginal delivery. Cesarean indications that were unspecified in the structured data were vaginal deliveries or not labeled in the structured data. Missing data for having received a transfusion (n=2) or induction were assumed to be not present. This was confirmed in a manual chart review. Four cases of “elective” cesarean section in the structured dataset were corrected after quality review by the delivering health care provider. In the existing, structured dataset, “unspecified” indications for cesarean section (n=120) were not labeled. Manual chart review resolved unlabeled cesarean indications in the physician-reviewed dataset (n=120). If the clinical notes listed more than one indication, the category was labeled as “other.” The datasets were normalized for multiple gestations for a single patient delivery encounter. All personal health information, dates, and demographic information were removed from the datasets. Having received a transfusion was selected as the target outcome. A transfusion was defined by one or more units of blood given.

A generalized linear model, random forest model, and XGBoost model were evaluated for performance. The XGBoost model was chosen due to its performance, internal cross-validation, and feature importance reporting. One-hot encoding was used to convert categorical variables. Cesarean section and use of induction were binary columns. The transfusion units column, which was numerical, was converted to a churn column. The transfusion units column was removed from the datasets prior to XGBoost model training to prevent data leakage. XGBoost was used in R (version 4.4.0; R Project for Statistical Computing). The model was trained with data split into 80% training and 20% testing for both datasets. Class imbalance was addressed using scale-positive weight and tune grid parameters in the *caret* package. Internal validation was performed with k-fold cross validation set to 5. Hyperparameter grid tuning and adjustment of maximum depth were performed. Models were compared for performance. The Youden *J* statistic threshold (sensitivity + specificity – 1) was calculated for predictions. The best-performing clinically relevant model was used in a prototype clinical decision support app for alpha testing (details in Multimedia Appendix 1). The TRIPOD +AI (Transparent Reporting of a Multivariable Prediction Model for Individual Prognosis or Diagnosis–Artificial Intelligence) checklist was used as a framework for model development (Multimedia Appendix 2 [16]).

**Table 1:** Demographics of the total delivery population (N=62,521).

Race			Values
**African American/Black**			
	**Age (years)**		
		Mean (SD)	27 (4.91)
		Range	50-13
	**Ethnicity, n (%)**		
		Total	7533 (12.05)
		Non-Hispanic	7371 (11.78)
		Hispanic	95 (0.15)
		Unknown	67 (0.11)
**Alaskan Native/American Indian**			
	**Age (years)**		
		Mean (SD)	29 (5.04)
		Range	40-17
	**Ethnicity, n (%)**		
		Total	55 (0.08)
		Non-Hispanic	41 (0.06)
		Hispanic	14 (0.02)
		Unknown	0 (0)
**Asian**			
	**Age (years)**		
		Mean (SD)	31 (5.04)
		Range	50-17
	**Ethnicity, n (%)**		
		Total	1184 (1.89)
		Non-Hispanic	1122 (1.79)
		Hispanic	40 (0.06)
		Unknown	22 (0.03)
**Multiracial**			
	**Age (years)**		
		Mean (SD)	27 (5.35)
		Range	48-13
	**Ethnicity, n (%)**		
		Total	1157 (1.85)
		Non-Hispanic	926 (1.48)
		Hispanic	172 (0.27)
		Unknown	59 (0.09)
**Native Hawaiian/Pacific Islander**			
	**Age (years)**		
		Mean (SD)	27 (5.05)
		Range	42-17
	**Ethnicity, n (%)**		
		Total	93 (0.14)
		Non-Hispanic	83 (0.13)
		Hispanic	6 (0.01)
		Unknown	4 (0.006)
**None of the above**			
	**Age (years)**		
		Mean (SD)	25 (4.08)
		Range	33-18
	**Ethnicity, n (%)**		
		Total	20 (0.03)
		Non-Hispanic	1 (0.002)
		Hispanic	19 (0.03)
		Unknown	0 (0)
**Other**			
	**Age (years)**		
		Mean (SD)	29 (4.55)
		Range	46-15
	**Ethnicity, n (%)**		
		Total	2936 (4.69)
		Non-Hispanic	1723 (2.75)
		Hispanic	1158 (1.85)
		Unknown	62 (0.09)
**Unavailable**			
	**Age (years)**		
		Mean (SD)	28 (5.49)
		Range	50-14
	**Ethnicity, n (%)**		
		Total	1269 (2.02)
		Non-Hispanic	311 (0.50)
		Hispanic	554 (0.89)
		Unknown	402 (0.64)
		Unavailable	2 (0.003)
**White/Caucasian**			
	**Age (years)**		
		Mean (SD)	28 (5.14)
		Range	53-13
	**Ethnicity, n (%)**		
		Total	48,274 (77.21)
		Non-Hispanic	47,362 (75.75)
		Hispanic	512 (0.82)
		Unknown	393 (0.63)
		None of the above	5 (0.008)

**Table 2:** Demographics of the study population (n=1734).

Race and ethnicity		Population, n (%)	Age (years), mean (SD)	Age range (years)
**African American/Black**				
	Non-Hispanic	308 (17.76)	26 (4.87)	43-15
	Hispanic	4 (0.23)	32 (2.43)	35-30
	Unknown	3 (0.17)	20 (3.55)	24-18
**Alaskan Native/American Indian**				
	Non-Hispanic	1 (0.06)	31 (0)	31-31
**Asian**				
	Non-Hispanic	31 (1.78)	31 (5.36)	41-19
	Hispanic	4 (0.23)	32 (0.49)	33-32
**Native Hawaiian**				
	Non-Hispanic	3 (0.17)	31 (1.18)	34-32
	Unknown	1 (0.05)	35 (0)	35-35
**Other**				
	Non-Hispanic	42 (2.42)	28 (6.19)	44-17
	Hispanic	57 (3.28)	26 (5.00)	41-18
**Multiracial**				
	Non-Hispanic	20 (1.15)	23 (6.25)	40-17
**Unknown**				
	Non-Hispanic	10 (0.57)	27 (5.85)	38-20
	Hispanic	17 (0.98)	26 (3.62)	35-22
	Unknown	15 (0.86)	29 (6.05)	41-20
**White/Caucasian**				
	Non-Hispanic	1215 (70.07)	26 (5.33)	48-13
	Hispanic	20 (1.15)	23 (4.34)	31-15
	Unknown	12 (0.69)	25 (7.06)	37-14

### Ethical Considerations

This study was reviewed by the Kettering Health Institutional Review Board and determined to be exempt from human subjects research requirements.

## Results

**Figure 1:**
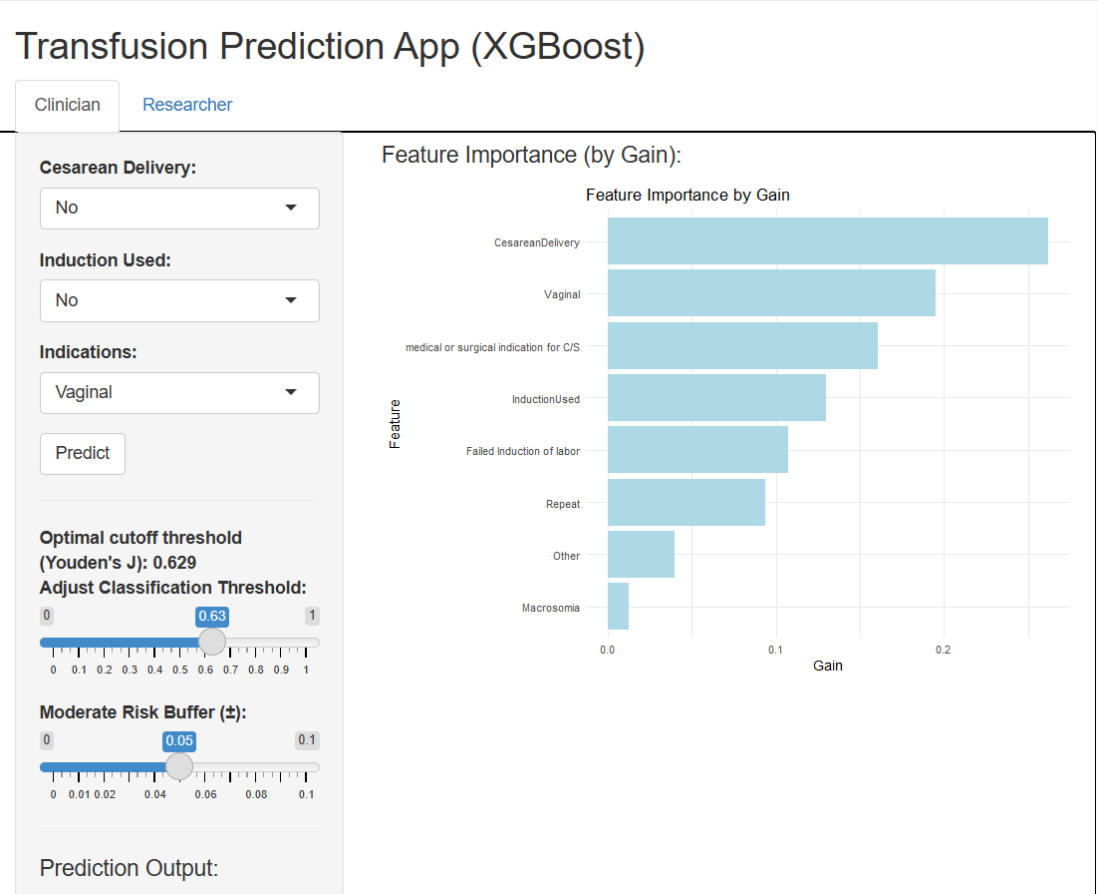
Transfusion prediction app clinician user interface for delivery mode, prediction output, and feature importance by gain.

**Figure 2:**
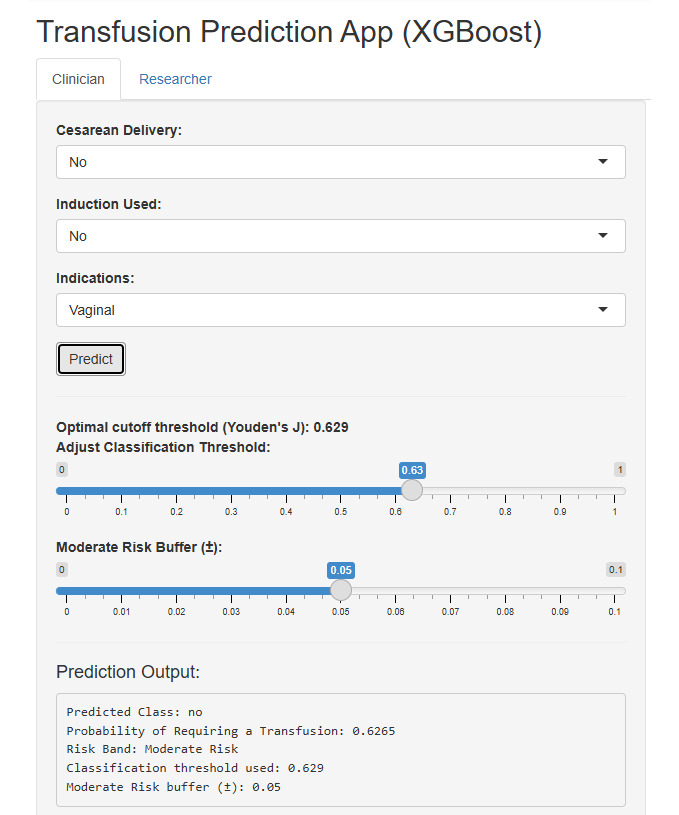
Transfusion prediction app clinician user interface prediction output and risk assessment (detail).

**Figure 3:**
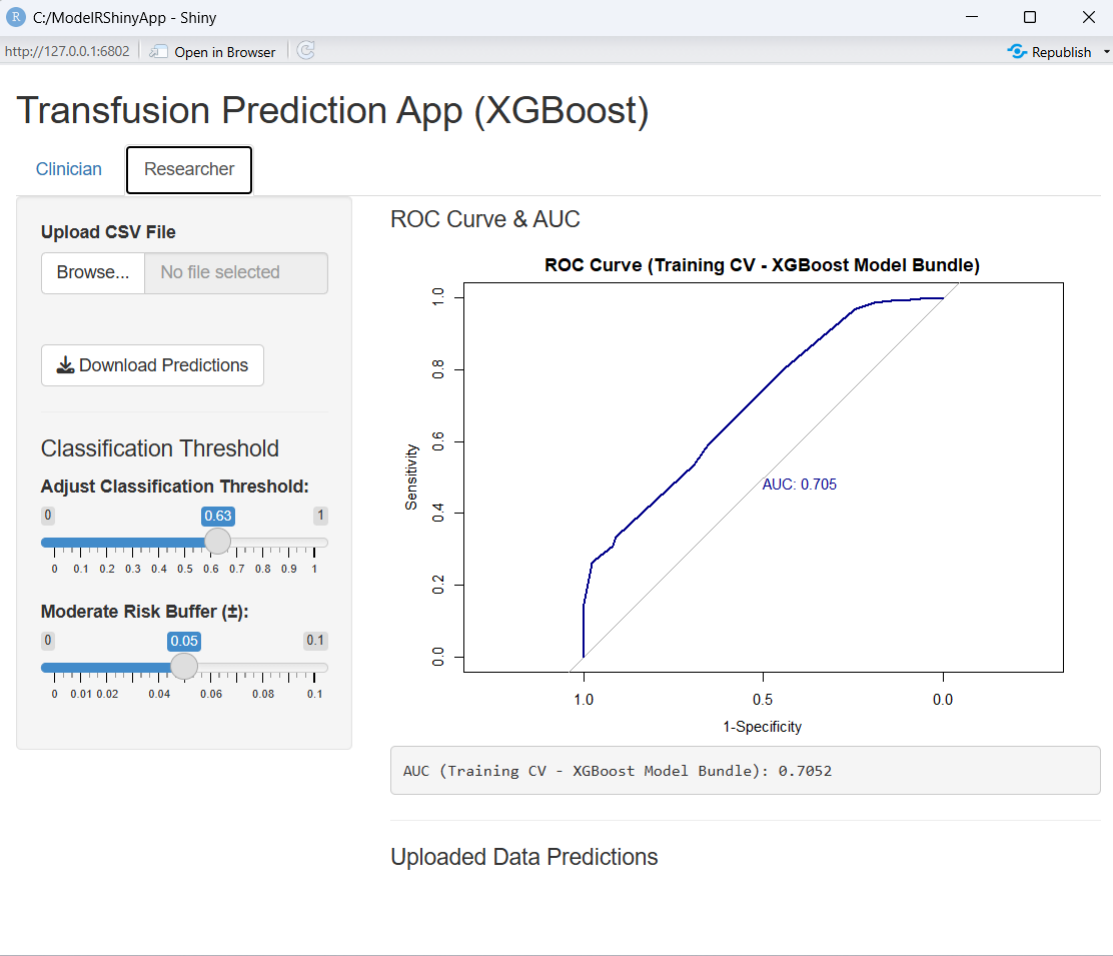
Transfusion prediction app researcher user interface with receiver operator characteristic plot, external dataset upload, and prediction download.

A generalized linear model, random forest model, and XGBoost model were evaluated for prediction of PPH transfusion. The XGBoost model had the best performance (receiver operating characteristic [ROC] curve=0.97), followed by the random forest (ROC curve=0.96) and generalized linear models (ROC curve=0.83). This was prior to removing the target transfusion units column. After dropping the target column, the XGBoost model using the existing structured data had the following metrics: area under the curve (AUC)=0.71, precision-recall ROC curve (PR-ROC)=0.82, and *F*_1_ score=0.805. The XGBoost model’s performance on the physician-reviewed dataset was as follows: AUC=0.705, PR-ROC=0.78, and *F*_1_ score=0.809. Feature importance by gain was vaginal delivery followed by cesarean section for medical or surgical indications for the existing, structured dataset. The most important feature for the physician-reviewed dataset was delivery mode. This was cesarean delivery, followed by vaginal delivery. The most important indication for a cesarean section was a medical or surgical indication (Figure 1). Alpha testing for app input (Figure 2) and dataset predictions were reviewed using the clinical app (Figure 3 and Multimedia Appendix 1).

## Discussion

### Principal Findings

Our model demonstrated that XGBoost was an accurate, clinically relevant predictor of transfusion (AUC=0.705; PR-ROC=0.78; and *F*_1_=0.809). This model had similar performance to the existing structured dataset (AUC=0.71, PR-ROC=0.82, and *F*_1_=0.80). The PR-ROC is a better metric for imbalanced datasets. Although the structured dataset had a higher PR-ROC of 0.82, the predictions and feature importance ranking were not clinically accurate. Model predictions for the structured dataset and the physician-reviewed dataset were comparable in performance for recall (0.97 vs 0.96), specificity (0.22 vs 0.25), and positive predictive value (0.69 for both). Accuracy was improved using the clinician-reviewed dataset (0.69 vs 0.708). However, a significant difference was noted in feature importance ranking and clinical predictions made with the clinical decision support app. The labeling of both cesarean sections and vaginal deliveries as “unspecified” indications for cesarean section affected the ranking of feature importance for the existing dataset. Thus, the dataset with existing EMR structured data did not give relevant clinical information to support a clinical decision tool. Explanation of model predictions and features are important for clinical support tools. Model explainability in this study was aided by interactive user interface outputs for low, moderate, or high risk stratification based on the threshold determined with the Youden *J* statistic (sensitivity + specificity – 1) [17]. (Figure 2; Multimedia Appendix 1) Researchers can upload external datasets for validation in the app. This feature allows predictions to be downloaded for review (Figure 3; Multimedia Appendix 3).

The XGBoost model was chosen for this study for its internal cross-validation, hyperparameter tuning, and feature importance ranking. Both datasets were evaluated with the same k-fold cross-validation (k=5). Hyperparameter tuning maximum depth was adjusted to improve model performance on the physician-reviewed dataset.

Model performance was compared for accuracy between an existing, structured dataset extracted from the EMR and a physician-reviewed dataset. Communication of transfusion rates and cesarean indication grouping prior to model training was an important aspect of data collection and feature selection. Physician engagement with data collection and labeling may help to explain the model’s predictions for future clinical use. Physician communication of the transfusion prevalence in our population and grouping of cesarean section indications led to development of the app. The physicians requested a clinical support tool that would help them discuss transfusion risk and delivery mode and fit into their clinical workflow.

In our study, delivery mode and medical or surgical indications for a cesarean section were important features. Delivery mode was one of the most predictive variables for PPH in the gradient boosting prediction model by Ahmadzia et al [7]. Despite an AUC of 0.83, the authors noted the model had difficulty predicting transfusion risk alone in a population with a 2.8% transfusion rate. Predicting transfusion risk in low-risk patients, however, remains challenging [18,19]. Many machine learning studies have sought to identify high-risk patients during labor. These studies have demonstrated machine learning to be useful in identifying high-risk populations compared to traditional risk-assessment tools [6,9,10,12,19].

Our clinical app was developed by physician request to aid clinical discussions of transfusion risk with a low, moderate, and high risk stratification. In this study, risk stratification was based on the Youden *J* statistic threshold (sensitivity + specificity – 1). This offers the clinician the ability to see how the model determines the classification. This display may improve patient and health care provider acceptance of the prediction. Physician input regarding the labeling and grouping of cesarean indications was important for data collection. Despite the promise of machine learning in PPH prediction, models designed for clinical use have yet to demonstrate utility in clinical practice. Clinicians use other machine learning–trained models to predict the success of a vaginal delivery after a cesarean section [20]. Thus, physicians may accept a clinical app to supplement discussions of PPH risk. It remains to be determined whether the model or app will generalize to other populations, improve patient outcomes, or be accepted by physicians and patients. Our model-supported app is a proof-of-concept approach to predicting PPH transfusion risk to facilitate shared decision-making. External validation is strongly recommended prior to app use in clinical settings. In addition, we recommend engaging physicians to participate in feature labeling to determine what features would be clinically meaningful to mitigate risk.

### Comparison to Prior Work

In our study, delivery mode followed by medical or surgical indications for a cesarean section were important features. As noted above, delivery mode was found to be the most predictive feature of PPH requiring a transfusion in the study by Ahmadzia et al [7]. Other studies have noted a 2% transfusion prevalence in their populations [3,12]. The low prevalence of transfusion in the study population and manual clinical data review pose resource challenges. For this reason, many studies use retrospective analysis of data to train models. Woo et al [11] found that large language models (LLMs) trained on clinical notes outperformed LLMs using structured data alone [11]. Clinically accurate information derived from LLMs may improve data quality, model development, and performance for future studies. LLMs trained on clinical notes may be a solution to obtain clinically accurate data in limited-resource and community settings.

Known risk factors for PPH were included in this study as indications for a cesarean delivery. High-risk conditions such as placental spectrum disorders, abruptio placenta, placenta previa, and HELLP (hemolysis, elevated liver enzymes, and low platelet count) syndrome were included in the medical or surgical indications for delivery. Prior PPH, grand multiparity, and prior cesarean section are high-risk conditions for PPH that would be known to the health care provider at the time of counseling for transfusion risk. In addition, risk factors that are unmodifiable prior to delivery such as age, BMI, race, and ethnicity were not included, as they would be known to the health care provider during counseling. Many studies have demonstrated machine learning models that can accurately predict PPH in high-risk conditions [6,8-10,12,18,21]. However, predicting PPH in low-risk patients remains challenging [9,18,19].

### Limitations

Limitations of this study include the retrospective nature of the review of records at a single community health system, the small sample size, the overlap of delivery indications, and the inability to demonstrate generalizability of the model with external validation.

### Future Directions

Further study is needed to determine if our proof-of-concept machine learning model–based clinical app will be generalizable and meaningful in clinical care settings. The external validation of our model is beyond the scope of this study.

### Conclusions

Our XGBoost model demonstrated accurate, clinically relevant prediction of transfusion (AUC=0.71, PR-ROC=0.78, *F*_1_ score=0.80) on structured data. The model performance on a physician-reviewed dataset was comparable (AUC=0.705, PR-ROC=0.78, and *F*_1_=0.809). Although these are similar metrics, the model gave more clinically appropriate predictions and feature importance ranking for the physician-reviewed dataset. For this reason, this model was selected for our proof-of-concept clinical decision support app. External validation is needed to see if this model will generalize to other populations. Further study is needed to determine if this app will be acceptable to patients and physicians as a useful prediction tool to improve patient care.

## Data Availability

A deidentified dataset and script are available through GitHub [22].

## Appendix

Multimedia Appendix 1 Prediction download from physician-reviewed dataset.

Multimedia Appendix 2 TRIPOD +AI checklist with page references.

Multimedia Appendix 3 Unreviewed dataset predictions.

## Abbreviations

AUC: area under the curve
EMR: electronic medical record
HELLP: hemolysis, elevated liver enzymes, and low platelet count
LLM: large language model
PPH: postpartum hemorrhage
PR-ROC: precision-recall receiver operating characteristic curve
ROC: receiver operating characteristic
TRIPOD +AI: Transparent Reporting of a Multivariable Prediction Model for Individual Prognosis or Diagnosis–Artificial Intelligence

## References

[1] (2019). Quantitative blood loss in obstetric hemorrhage: ACOG committee opinion, number 794. Obstet Gynecol.

[2] Committee on Practice Bulletins-Obstetrics (2017). Practice bulletin no. 183: postpartum hemorrhage. Obstet Gynecol.

[3] Venkatesh K, Strauss R, Grotegut C, Heine R Philip, Chescheir Nancy C, Stringer Jeffrey S A, Stamilio David M, Menard Katherine M, Jelovsek J Eric (2020). Machine learning and statistical models to predict postpartum hemorrhage. Obstet Gynecol.

[4] Mehrnoush V, Ranjbar A, Farashah MV, Darsareh F, Shekari M, Jahromi MS (2023). Prediction of postpartum hemorrhage using traditional statistical analysis and a machine learning approach. AJOG Glob Rep.

[5] (2019). ACOG Committee opinion no. 761: cesarean delivery on maternal request. Obstet Gynecol.

[6] Albright CM, Spillane TE, Hughes BL, Rouse DJ (2019). A regression model for prediction of cesarean-associated blood transfusion. Am J Perinatol.

[7] Ahmadzia HK, Dzienny AC, Bopf M, Phillips JM, Federspiel JJ, Amdur R, Rice MM, Rodriguez L (2024). Machine learning models for prediction of maternal hemorrhage and transfusion: model development study. JMIR Bioinform Biotechnol.

[8] Akazawa M, Hashimoto K, Katsuhiko N, Kaname Y (2021). Machine learning approach for the prediction of postpartum hemorrhage in vaginal birth. Sci Rep.

[9] Lengerich BJ, Caruana R, Painter I, Weeks WB, Sitcov K, Souter V (2024). Interpretable machine learning predicts postpartum hemorrhage with severe maternal morbidity in a lower-risk laboring obstetric population. Am J Obstet Gynecol MFM.

[10] Kawakita T, Mokhtari N, Huang J, Landy H (2019). Evaluation of risk-assessment tools for severe postpartum hemorrhage in women undergoing cesarean delivery. Obstet Gynecol.

[11] Woo E, Zighelboim I, Gifford T, Bell Joseph G, Milthorpe Hannah, Alsentzer Emily, Longman Ryan E, Tolosa Jorge E, Beaulieu-Jones Brett K (2025). Predicting postpartum hemorrhage using clinical features extracted with large language models. O G Open.

[12] Westcott Jill M, Hughes Francine, Liu Wenke, Grivainis Mark, Hoskins Iffath, Fenyo David (2022). Prediction of maternal hemorrhage using machine learning: retrospective cohort study. J Med Internet Res.

[13] Aghajanian S, Jafarabady K, Abbasi M, Mohammadifard F, Bakhshali Bakhtiari M, Shokouhi N, Saleh Gargari S, Bakhtiyari M (2024). Prediction of post-delivery hemoglobin levels with machine learning algorithms. Sci Rep.

[14] Baeta T, Rocha ALL, Oliveira JA, Couto da Silva AP, Reis ZSN (2025). Accuracy of machine learning and traditional statistical models in the prediction of postpartum haemorrhage: a systematic review. BMJ Open.

[15] Shah SY, Saxena S, Rani SP, Nelaturi N, Gill S, Tippett Barr B, Were J, Khagayi S, Ouma G, Akelo V, Norwitz ER, Ramakrishnan R, Onyango D, Teltumbade M (2023). Prediction of postpartum hemorrhage (PPH) using machine learning algorithms in a Kenyan population. Front Glob Womens Health.

[16] (2024). TRIPOD+AI statement: updated guidance for reporting clinical prediction models that use regression or machine learning methods. BMJ.

[17] Schisterman EF, Faraggi D, Reiser B, Hu J (2008). Youden Index and the optimal threshold for markers with mass at zero. Stat Med.

[18] Vasudevan L, Kibria MG, Kucirka LM, Shieh K, Wei M, Masoumi S, Balasubramanian S, Victor A, Conklin JL, Gurcan MN, Stuebe AM, Page D (2025). Machine learning models to predict risk of maternal morbidity and mortality from electronic medical record data: scoping review. J Med Internet Res.

[19] Yao X, Bao Y, Wu N, Shan S, Xu Y, Huo K, Huang R, Ying H (2025). Interpretable machine-learning-based prediction of postpartum haemorrhage in normal vaginal births in Shanghai, China. Front Med (Lausanne).

[20] Metz TD, Stoddard GJ, Henry E, Jackson M, Holmgren C, Esplin S (2013). Simple, validated vaginal birth after cesarean delivery prediction model for use at the time of admission. Obstet Gynecol.

[21] Neary C, Naheed S, McLernon D, Black M (2021). Predicting risk of postpartum haemorrhage: a systematic review. BJOG.

[22] OB-Transfusion-App-Prototype. GitHub.

